# A Network-Based Multi-Target Computational Estimation Scheme for Anticoagulant Activities of Compounds

**DOI:** 10.1371/journal.pone.0014774

**Published:** 2011-03-22

**Authors:** Qian Li, Xudong Li, Canghai Li, Lirong Chen, Jun Song, Yalin Tang, Xiaojie Xu

**Affiliations:** 1 Beijing National Laboratory for Molecular Sciences, State Key Lab of Rare Earth Material Chemistry and Applications, College of Chemistry and Molecular Engineering, Peking University, Beijing, People's Republic of China; 2 Beijing National Laboratory for Molecular Sciences, Center for Molecular Sciences, State Key Laboratory for Structural Chemistry of Unstable and Stable Species, Institute of Chemistry Chinese Academy of Sciences, Beijing, People's Republic of China; 3 Experimental Research Center, China Academy of Chinese Medical Sciences, Beijing, People's Republic of China; 4 Graduate University of Chinese Academy of Sciences, Beijing, People's Republic of China; German Cancer Research Center, Germany

## Abstract

**Background:**

Traditional virtual screening method pays more attention on predicted binding affinity between drug molecule and target related to a certain disease instead of phenotypic data of drug molecule against disease system, as is often less effective on discovery of the drug which is used to treat many types of complex diseases. Virtual screening against a complex disease by general network estimation has become feasible with the development of network biology and system biology. More effective methods of computational estimation for the whole efficacy of a compound in a complex disease system are needed, given the distinct weightiness of the different target in a biological process and the standpoint that partial inhibition of several targets can be more efficient than the complete inhibition of a single target.

**Methodology:**

We developed a novel approach by integrating the affinity predictions from multi-target docking studies with biological network efficiency analysis to estimate the anticoagulant activities of compounds. From results of network efficiency calculation for human clotting cascade, factor Xa and thrombin were identified as the two most fragile enzymes, while the catalytic reaction mediated by complex IXa:VIIIa and the formation of the complex VIIIa:IXa were recognized as the two most fragile biological matter in the human clotting cascade system. Furthermore, the method which combined network efficiency with molecular docking scores was applied to estimate the anticoagulant activities of a serial of argatroban intermediates and eight natural products respectively. The better correlation (r = 0.671) between the experimental data and the decrease of the network deficiency suggests that the approach could be a promising computational systems biology tool to aid identification of anticoagulant activities of compounds in drug discovery.

**Conclusions:**

This article proposes a network-based multi-target computational estimation method for anticoagulant activities of compounds by combining network efficiency analysis with scoring function from molecular docking.

## Introduction

The formation of a fibrin clot at the site of an injury to the wall of a blood vessel is an essential part in stop blood loss after vascular injury. The reactions that lead to the formation of fibrin clots are commonly described as the clotting cascade, in which the product of each step is an enzyme or cofactors necessary for the following reactions to proceed effectively[1]. The clotting cascade can be divided into three parts, the extrinsic pathway, the intrinsic and the common pathway[2]. The extrinsic pathway begins with the release of tissue factor at the site of vascular damage and leads to the activation of factor X. The route provides an alternative mechanism to activate factor X, from the activation of factor XII. The common pathway is composed of steps linking the activation of factor X to the formation of a multimeric, cross-linked fibrin clot. Each of these processes includes not only a cascade of events that generate the necessary catalyst for the formation of clots, but also many positive and negative regulatory events.

As a result of advances of computational techniques and hardware solutions, virtual screening has dramatically speeded up modern lead identification and lead optimization. Ligand-based and structure-based virtual screening are two most important methods used in current computer aided drug design[3]. Ligand-based methods such as chemical similarity analysis[4] and pharmacophore modeling[5] mainly focused on the features of the active ligands structure. With high performance output, ligand-based virtual screening was widely used to screen large compound database. However, the fundamental problem of the methods is that definition of what constitutes an active scaffold is highly subjective[6]. Synergized with X-ray crystallography, NMR spectroscopy and isothermal titration calorimetry (ITC), structure-based virtual screening has been used to complement experimental high-throughput screening (HTS) methods to improve the efficiency and efficacy of discovering lead inhibitors[7]–[11]. Structure-based screens typically the molecular docking to fit small organic molecules into targets of known structure, evaluate them for structural and chemical complementary. In last few years, investigators have also turned to predict new substrates for enzymes or receptors of unknown function (such as the membrane proteins) and to predicting potent small molecules based on multi-targets.

With emergences of new paradigms in multi-target drug discovery for several complex diseases, multi-target virtual screening has been presented and executed to discover the regimen which could target many different proteins and could be of low cost, efficacy and better tolerance. However, the importance and role of target in many complex disease systems were not explicitly considered in the reported literatures about multi-target virtual screening. Moreover, as most traditional virtual screening method, more attention was paid on binding affinity between drug molecule and target instead of phenotypic data of drug molecule against disease system[12].

**Figure 1:**
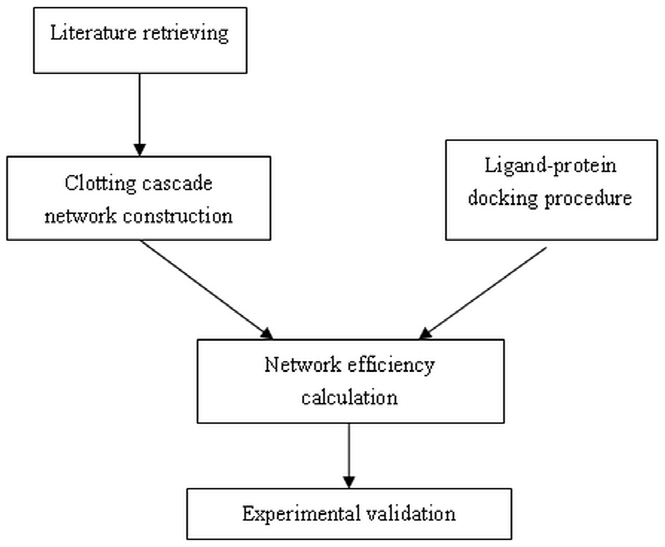
The work flow of our virtual screening approach.

With the progress of system biology and bionetwork, we know that the biological potency of an ideal drug may not merely determined by the inhibition of a single target, but rather by the rebalancing of several proteins or events, which contribute to the etiology, pathogeneses, and progression of a complex disease [13]–[26]. The available methodologies of in silico screening based on a single target seem not effective in studying ligands' effects on biological process comprehensively for some cases[27], [28]. In the current work, a novel approach was developed by integrating the predictions based on multi-target docking studies through biological network efficiency analysis to estimate the biological potency[26], [29]–[31]. The work flow was shown in Figure 1. The satisfactory predictions of our model were validated by the experiments. Similar model to predict the biological potency of drugs quantitatively by combining the multi-target virtual screening and biological network calculation together have not been yet reported in the past references. This novel model could be a powerful tool for combinatorial drug discovery and the development of multi-target drugs.

## Methods

### 1 Constructions of the docking library

The docking library for multi-target virtual screening against clotting cascade comprises 1177 compounds from 24 Traditional Chinese Medicines (TMCs) that were widely used as components of recipes against cardiac system diseases. These TCMs include 23 original plants and 1 original animal (their information can be found in the Supporting Information S1). All compounds identified in these TCMs were collected from Chinese Herbal Drug Database developed in our group[32] and other literatures[5], [33]–[35]. In addition, some active synthetic compounds against coagulation cascade available to our laboratory were included in the docking library, for example, seven argatroban intermediates. The structures of these compounds were constructed and minimized with the MMFF force field[36] in Discovery Studio molecular simulation system (DS, Accelrys Inc.). In minimization, the threshold of root mean square deviation (RMSD) of potential energy was set to 0.001 kcal·Å-1·mol-1. The optimized structures of all compounds were saved as sdf and mol2 formats, respectively, for further docking study and were included in the Supporting Information S1.

### 2 Network-Based dual-step hierarchical Computational Estimation

Fourteen proteins authorized as drug targets by US Food and Drug Administration (FDA) were used in the virtual screening based on docking simulations. These targets include coagulation factor Xa, thrombin, coagulation factor IXa, tissue factor:coagulation factor VIIa complex, coagulation factor VIIa, fibrin, kallikrein, tissue factor, prothrombin, von Willebrand factor, coagultaion factor VIII, coagulation factor XI, fibrinogen, and coagulation factor XIII. To reduce computational cost while not degrade the calculation accuracy, two docking approaches, including Ligandfit and Autodock, were successively employed to dock candidates to the binding sites of these receptors in accordance with the order of their docking simulation accuracies in network-based dual-step hierarchical virtual screening. Top ten percent of hits from the previous step were used for the next step. In every steps of serial virtual screening, one candidate was estimated and ranked based on its influence on the network efficiency of clotting cascade network instead of the scoring functions of these binding poses on one target as used in conventional virtual screening methods.

#### (a) Docking and scoring with Ligandfit

The crystal structures of fourteen targets were retrieved from the Protein Data Bank (PDB entries: 1FJS[37], 1TA2[38], 1RFN[39], 1W0Y[40], 1YGC[41], 2HLO, 2ANW[42], 1TFH[43], 1K22[44], 1AUQ[45], 3CDZ[46], 2F83[47], 1FZG[48] and 1GGT[49]). Hetero atoms were removed from the receptors, and then hydrogen atoms were added and wrong valence shells were corrected using Discovery Studio. For receptor/ligand complex with crystal structure, the binding site was defined as the grid points around the ligand which were unoccupied by receptor atoms, whereas for a receptor without crystal complex structure, potential binding sites were found based on the shape of the receptor. Ligandfit protocol in Discovery Studio was used to dock ligands into the specified site by the following steps: (1). conformational search of candidate ligand for docking, (2). ligand/site shape matching, (3). positioning the selected ligand conformation into the binding site, and (4) rigid body energy minimization of the candidate ligand pose/conformation using the DockScore energy function and updating the saved list of ligands with the candidate pose. Except maximum poses retained was set to 1, and default values were adopted for the other parameters. The Piecewise Linear Potential 1 (PLP1) was selected for subsequent calculation of network efficiency shift of all compounds based on our previous work about the comparison of several empirical scoring functions[50].

#### (b) Docking and Scoring with Autodock

The AutoDock4.01 program was used for the second step of the dual-step hierarchical virtual screening because of the better performance of its scoring function over those of the others for several target proteins[51]. First, polar hydrogen atoms were added and non-polar hydrogen atoms were merged by the Hydrogen module in AutoDock Tools (ADT) for fourteen targets after water molecule were removed. Then, Kollman united atom partial charges were assigned. The grid map of the docking simulation was established by a 61×61×61 cube centered on the target active site as defined in Ligandfit, with a spacing of 0.375 Å between the grid points. When every ligand was docked to a target, the Lamarckian genetic algorithm were used optimize the conformation of ligand in the binding pocket. The set of parameters was listed as following: the size of the population was 150. The number of energy evaluations was set to 1.75×10^7^ as the run terminates. For clustering the conformations, the root mean square deviation tolerance was 2.0. Twenty independent docking runs were carried out for every ligand. Other parameters were set to default. For the targets of which crystal complex structures were determined, every ligand in complexes was picked up and sequentially docked back into its initial active sites respectively in order to assess the reliability and accuracy of docking by Autodock program.

### 3 Network construction and analysis

#### (a) Network construction

The network was constructed using the information from Reactome knowledgebase[52]. The clotting cascade pathway has been chosen to build the network. The enzymes which participate in the pathway were proposed as nodes and arrows between nodes represent the connections. The direction of the arrow means that the enzyme in the end of the arrow enhances the formation of the enzyme located in the front.

#### (b) Network statistics

The damage induced by the attacks on the network is characterized by the network efficiency (NE), which is defined as the sum of the reciprocals of the shortest path lengths between all pairs of nodes[53]. Due to a global topological property of a network which could be applied to measure the integrity of the network, the network efficiency was assumed to be used as a measure for drug efficiency[54]. The NE of a graph G is measured by the shortest paths between pairs of nodes with the expression:
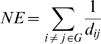
where dij is the length of the shortest path between node i and j and the sum is over all N(N −1)/2 pairs of nodes with total number N of nodes in the graph G. If the network is weighted, dij is the path with the minimum weight. The initial line values of every edge were arbitrarily set to 10. To give relative network efficiency, this quantity NE is divided by the initial network efficiency. Thus we considered the network efficiency of the initial network as 100% and measured the relative network efficiency after each attack. We have chosen the clotting cascade network as the network models.

#### (c) Network efficiency calculation

The network efficiency was calculated for each compound. The compounds' effects to the network rely on the docking scores. We supposed that the compound could inhibit the target well while the docking scores were relatively high. For a ligand, we transformed its docking scores with a target to line values of all directly downstream edges of the target in the network and then calculated the network efficiency. In other word, the line values of all edges, which point to the other targets from this target, were re-assigned based on the docking score between the compound and the target. The docking score threshold was set to 0, so any docking score which was positive was fixed to 0. For each docking target, the ligand with the highest binding energy was chosen as the reference standard. We defined that the most potent ligand would knock the target by 99.95%. Therefore, the ligand could make the value of the lines that come out of the target enzyme as 200. As the reference ligand docking score should make the line values to 200, the factor 2.3 was used to achieve this purpose. That was because the 2.3th power of 10 is equal to 200 and that was where the factor 2.3 come out from. Therefore, the ligand could make the value of the lines that come out of the target enzyme as 200. BEs represents the binding free energy of the most potent ligand, BE represents the binding free energy of other ligands, and LV is the line values of the edges come out of the target in the network. The line values of the edges which did not come out of the target enzyme were defined as 10. The line values of the edges in the network were calculated with the expression:
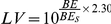
Therefore, different ligand would show different effect on the target. For each ligand the network efficiency was then recalculated using the redefined line values. The network efficiency of each ligand was ranked by the decrease of the network efficiency. The more the network efficiency decreases, the more potent the ligand is. The procedure of network efficiency calculation was written in C language using Dijkstra Algorithm.

### 4 Experimental validations

Among these compounds, we chose fourteen compounds which could be purchased for the further experimental validations. The compounds used in the experiments were: fangchinoline, folic acid, rutin, quercetin, liensinine, salvianolic_acid_A, salvianolic_acid_B and six argatroban intermediates. The structures of these compounds were showed in the table 1.

**Table 1:** Results of clotting assays and network efficiency.

Test compounds	ΔAPTT ratio	ΔPT ratio	ΔTT ratio	sum	Decrease of Network efficiency
salvianolic acid a	0.121	0.175	0.296	0.592	11.78
salvianolic acid b	0.375	0.132	0.265	0.771	11.97
rutin	0.057	0	0.783	0.84	12.69
quercetin	0	0.122	0.455	0.577	11.48
liensinine	0.206	0	0.113	0.319	11.42
fangchinoline	0.215	0.022	0.161	0.398	11.62
folic acid	0.268	0.007	0.167	0.442	10.96
L-glutamine	0.068	0.098	0.035	0.2	8.74
Argatroban intermediate 1	0.384	0.065	0.112	0.56	10.63
Argatroban intermediate 2	0.368	0.049	0.182	0.599	10.26
Argatroban intermediate 3	0.243	0.081	0.126	0.45	11.47
Argatroban intermediate 4	0.305	0.138	0.161	0.604	11.94
Argatroban intermediate 5	0.167	0.033	0.196	0.395	10.9
Argatroban intermediate 6	0.294	0.122	0.231	0.648	12.05

Biological activity results of relative APTT (Activated Partial Thromboplastin Time), PT (Prothrombin Time) and TT (Thrombin Time), sum of the three ratios of times and calculated decrease of network efficiency after treated of fourteen compounds. The relative ratios were calculated by the sample time minus the relevant vehicle control time and then divided by the relevant vehicle control time.

Activated partial thromboplastin time (aPTT), prothrombin time(PT) and thrombin time(TT) assays were performed using a model LG-PABER-I coagulometer (Steellex Scientific Instrument Company, which also provided the used plasma and clotting reagents) in compliance with manufacturers' recommendations. All test chemicals with the exception of heparin sodium were solutized and subsequently serial ten-fold dilutions were prepared with dimethyl sulfoxide (DMSO) to yield a range of concentrations (12,1.2,0.12,and 0.012×10-3 moles·L-1). Heparin sodium solutions (9.0×104 I.U.·L-1) were prepared by dissolving in normal saline (NS). The above series of solutions (DMSO and NS as references) were diluted with pooled normal human plasma (1∶60 vol:vol), and the mixed plasma were evaluated with aPTT, PT and TT values, which associate with anticoagulant potential of test chemicals. All reactions were performed in duplicate and data is expressed in clot time (second).

## Results and Discussions

**Figure 2:**
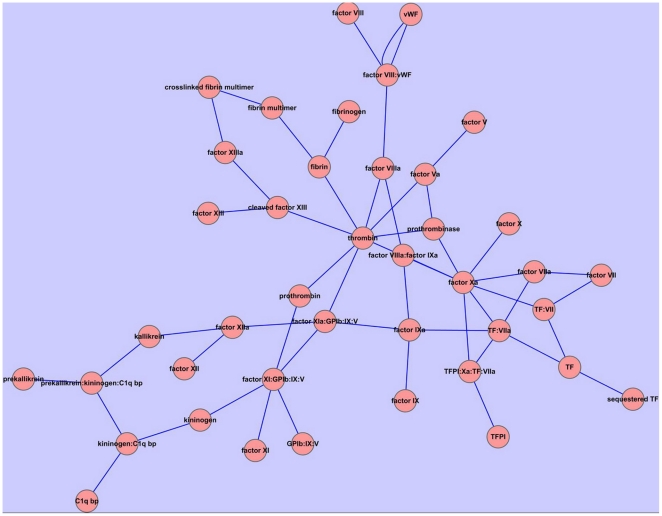
The network constructed according to the clotting cascade pathway. The red nodes represent the enzymes participate pathway and the lines between the nodes reflect the relationships between the enzymes of the clotting cascade pathway. The network contained 41 nodes (enzymes) and 55 edges (relationships between enzymes).

The constructed network (Figure 2) contains 41 nodes and 53 edges (arrows). The nodes cover most of the important enzymes that participate in the clotting cascade, such as thrombin, factor X, factor V, TF, etc. We removed each node and calculated the network efficiency to determine the importance of the enzyme. The results show that the nodes corresponding to factor Xa, thrombin and factor VIIIa:factor IXa are identified as the top three critical targets. The deletion of factor Xa could reduce the network efficiency greatly, from 17.822 to 8.894. And knock out of thrombin from the network could reduce the network efficiency from 17.822 to 10.542. Our predictions are in good agreement with the reported results[55], [56]. For example, the approved drug Arixtra is a synthetic and specific inhibitor of activated factor X (Xa) indicated for the prophylaxis of deep vein thrombosis, which may lead to pulmonary embolism. That means the inhibition of factor Xa is an ideal way for thrombosis treatment, which is consistent with our prediction. As a crucial role in physiological and pathological coagulation, thrombin can be considered a very successful drug target because numerous direct thrombin inhibitors, e.g., Hirudin, Bivalirudin, Lepirudin, Desirudin, Argatroban, Melagatran and Dabigatran are in clinical use or undergoing clinical development as antithrombosis agents[57]. In order to test the clotting cascade network, we randomly deleted one enzyme in the network and compute the correspondence network efficiency. Deletion of enzyme with most network efficiency drop could be considered as the most important targets. The results showed that deletion of thrombin and Factor Xa would take most effect to the network efficiency. That meant thrombin and Factor Xa were predicted as the most important targets by network efficiency calculation. This prediction was in accordance with practical knowledge. Therefore, our clotting cascade network could mainly reflect the real biological process. After the clotting cascade network testing, docking validations also should be carried out.

To quantitatively compare the differences of the positions and orientations of five ligands from targets with complex structures between experimental and computational conformations, all RMSD (root mean square deviation) between experimental and computational conformations of these ligands in these complexes were calculated. RMSD of coagulation factor Xa, thrombin, prothrombin and tissue factor/factor VIIa are 1.811, 1.890, 1.702 and 1.943, respectively. Among all five values, only RMSD of factor VIIa is larger than 2 Å and is 2.489, but it is acceptable after analyzing the positions and orientations of functional groups in the ligand. (The details are stated in the Supporting Information S1.) These results indicate that docking by Autodock program in our study is reliable and accurate enough for further analyses.

In the experimental section of this study, three clinical used blood clotting assays: aPTT, PT and TT were carried out to reveal the biological activities of the 14 test compounds. The activated partial thromboplastin time (aPTT) mainly reflects the intrinsic pathway which is part of the clotting cascade. The prothrombin time (PT) is measure of the extrinsic pathway of coagulation. The Thrombin Time (TT), is a blood test which measures the time it takes for a clot to form in the plasma from a blood sample in anticoagulant which had added an excess of thrombin. However, the three assays could not correlate to any single-target. As the three assays individually reflect part of the clotting cascade, we considered sum of the three experimental measurements could represent the whole effects of clotting cascade. Therefore, we correlate the network efficiency to the sum of the three experimental measurements.

Then, we conducted multi-target docking for fourteen compounds. Each compound was initially docked and then ranked by the predicted binding energy to obtain the line values in the network. After that, the network efficiency for each compound was calculated. We compared the performances of two docking approaches, Autodock and Ligandfit, and found that the decrease of the network deficiency based on the predictions given by Autodock can give better correlation with the experimental data (r = 0.671) than that based on the predictions given by Ligandfit (r = 0.47).

In order to test the probability for large scale screening of this method, we evaluated the runtime of the docking approach and the network efficiency calculation which were the most time consuming procedures in this method. Take this study for example, docking procedures for one compound mainly cost less than 10 minutes per target per CPU thread. Runtime of network efficiency calculation was relay on the number of targets in the network. The runtime complexity for the worst-case scenario of the network efficiency calculation is O(n^2^) while the “n” is the number of targets in the network. The network size could be extended to hundreds of nodes and thousands of edges. That would large enough for current disease pathway. For the typical 14 target network in this study, calculating the network efficiency for 14 compounds based on docking data only cost less than 2 seconds on a single CPU core. If our method was running on a cluster which contain 64 CPUs (128cores, 256 threads), the throughput could attain to more than 30 000 compounds per day. Therefore, it is a feasible approach for large scale screening.

**Figure 3:**
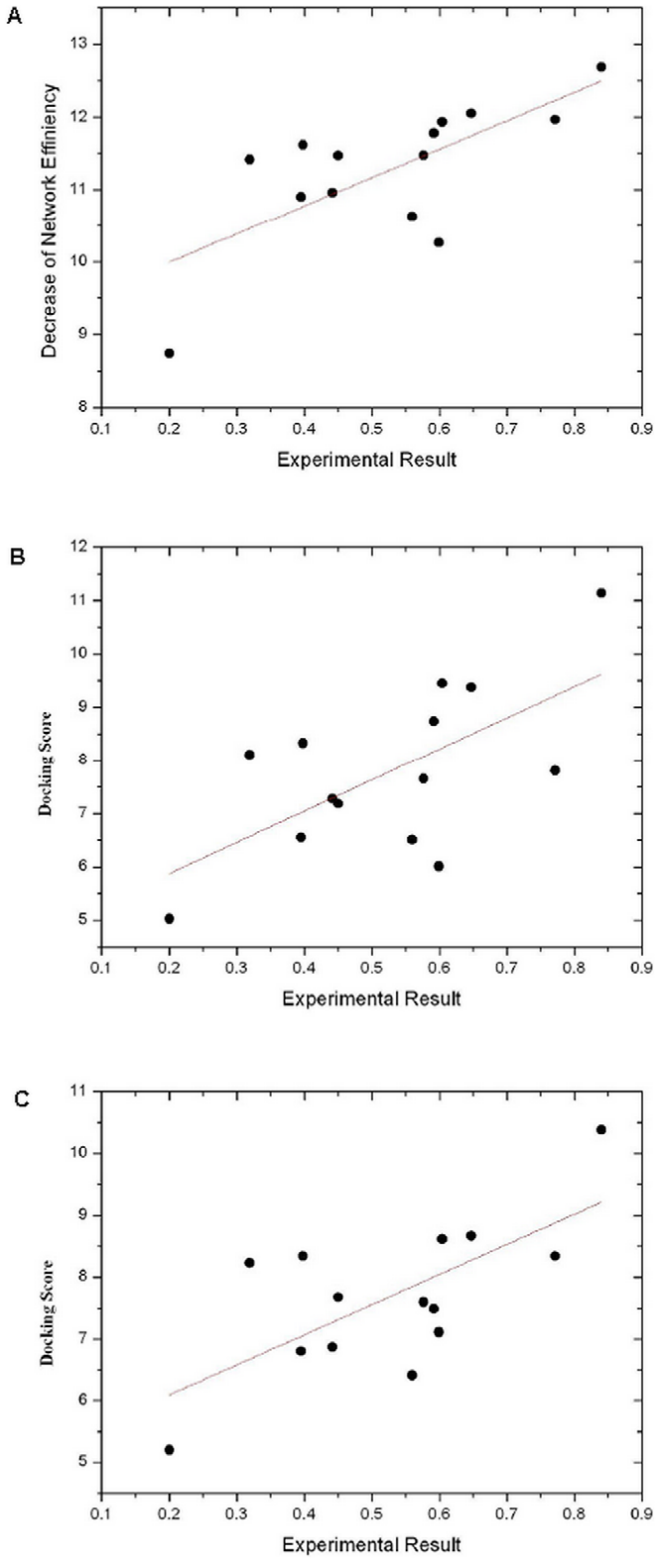
Comparison the predicting ability of the network-based multi-target computational estimation scheme with single-target docking scoring function. A) The correlation (r = 0.671) between the integrated fourteen compounds biological activities and the decreases of network efficiency induced by these compounds. The decrease of network efficiency is calculated from the multi-target docking scoring. B) The correlation (r = 0.648) between the fourteen compounds biological activities and the docking scores with coagulation factor Xa. C) The correlation (r = 0.602) between the fourteen compounds biological activities and the docking scores with thrombin. The biological activities of the fourteen compounds are illuminated in the Supporting Information S1.

To reveal the importance of the network efficiency, we compared the predictions by the network efficiency analysis based on the single target docking and those based on the multi-target docking. When computed by applying the single target docking scores, the correlation coefficients of the estimated potency and the experimental data for factor Xa and thrombin, which are supposed as very important enzymes in clotting cascade[56], [58], [59], [60], were 0.648 and 0.602(Figure 3B and Figure 3C), respectively. However, the correlation between the predicted network efficiencies and the experimental data was improved by applying the multi-target docking scores (r = 0.671) (Figure 3A). It suggests that overall consideration of the contribution of the biological network might be better than only consideration of the contribution of single target for the accurate predictions of the biological activities. The single target docking cannot capture the biological effects of the ligands comprehensively, and the multi-target docking is really necessary to characterize the complicated binding process of ligands with multiple targets involved in biological network.

**Figure 4:**
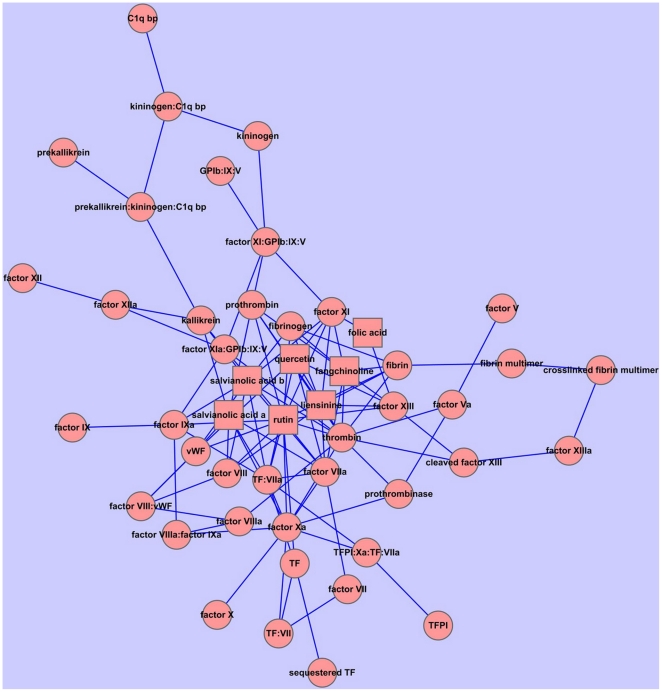
The Drug-Target network. Circles represent the enzymes in the clotting cascade pathway and the boxes represent the hit compounds (rutin, salvianolic acid a, salvianolic acid b, fangchinoline, quercetin, liensinine, folic acid). Each ligand is assumed to connect with its target if it can form strong interactions with the target. Their interactions are expressed by the connecting edges.

Additionally, we analyzed the potency of the hit compounds (In order to emphasis on the hit compounds screening from Traditional Chinese Medicine, six argatroban intermediates were not include in the analysis.) through the network connectivity. In Figure 4, a ligand is assumed to connect with its target if it can form strong interactions with the target. The compound rutin, which connects with 14 targets, is the most potent compound according to our experiments. Other hit compounds, such as liensinine and folic acid, which have less connecting neighbors, show limited biological activities. It seems that the compounds which can connect with more targets have higher activities because the potent compounds interact not only with a single target but also with a series of important targets in the clotting cascade pathway. Therefore, the technique which combines multi-target docking and biological pathway network analysis can predict the effects of ligands to the whole biological pathway more efficiently.

An analysis of the pharmacology literature was used to assess the whole homeostasis property of the compound with a larger decrease value in network efficiency. Previous reports[61], [62] suggested that rutin protected stroke and inhibited thrombosis. Salvianolic acid B is also confirmed effective on modulating hemostasis properties of human umbilical vein endothelial cells[63]. Salvianolic acid A is found protective against cerebral and myocardial ischemia and reperfusion[64]. These findings indicate that network efficiency analysis combined with molecular docking scoring function can be used to successfully screen natural product databases of potential drugs in silico to identify molecules with anticoagulant activity.

Generally speaking, current virtual screening methods mainly focus on single drug-target interaction. The correlation coefficients between the estimation and the experiment values were based on compounds' effects of single target inhibition. However, compounds' effects on single target inhibition hardly correlated to the whole effects on such biological pathway process. At the same time, a few other studies also tried to relate drug effects via pathway alterations. Mitsos et al. have described a phosphoproteomic-based approach to identify drug effects by monitoring drug-induced topology alterations[65]. They started with a generic pathway made of logical gates and performed fitting via an Integer Linear Program (ILP) formulation. While in our study, we constructed our screening network based on clotting cascade and applied the Network Efficiency (NE) for ligand efficiency prediction. Herein, this method reflected the compound's effects on the biological pathway and correlated to the phenotype data which could provide different opinions on pathway based virtual screening.

Like all virtual screening scoring method, our approach has many advantages as well as some limitations. One of obvious advantages of the method is that it specifically considers the role of every target in the whole coagulation cascade process and assigns the weightiness on every target by biological network analysis. The other advantage is that the affinity evaluation in the method is not limited to molecular docking and scoring, as used in this study. Other binding energy prediction methods could also be used, such as pharmacophore, quantitative structure-activity relationship or comparative molecular field analysis. It is also assumed that the consideration of flexibility of the targets in molecular docking might improve the accuracy of the network efficiency. The relevant work how flexible docking and precise binding free energy computational methods affect the accuracy of the network efficiency is under way. Given the fact that the x-ray structures of fourteen enzymes in existing networks have been determined, molecular docking and scoring function are well suited for the human coagulation cascade system. A clear disadvantage of this technique is that its accuracy enormously depends on the reliability of network construction and the veracity of binding affinity assessment.

In summary, we developed a model that combines multi-target docking and network efficiency calculation for the predictions of the potency of ligands with reasonable accuracy. The method integrates the scores given by the multi-target docking scores by the network efficiency analysis according to the targets' importance in a biological pathway or process. The network efficiency analysis based on the multi-target docking can evaluate the ligands' potency more comprehensively than the traditional single target docking and show better prediction accuracy. It remains to be determined how the size and complexity the biological network take effect to the biologically relevant, and the relevant work is under way.

## Supporting Information

Supporting Information S1 (0.23 MB DOC)

## References

[1] Mann KG, Butenas S, Brummel K (2003). The dynamics of thrombin formation.. Arterioscler Thromb Vasc Biol.

[2] Davie EW, Fujikawa K, Kisiel W (1991). The Coagulation Cascade - Initiation, Maintenance, and Regulation.. Biochemistry.

[3] Rester U (2008). From virtuality to reality - Virtual screening in lead discovery and lead optimization: a medicinal chemistry perspective.. Curr Opin Drug Discov Dev.

[4] Bender A, Mussa HY, Glen RC, Reiling S (2004). Similarity searching of chemical databases using atom environment descriptors (MOLPRINT 2D): evaluation of performance.. J Chem Inf Comput Sci.

[5] Sun H (2008). Pharmacophore-based virtual screening.. Curr Med Chem.

[6] Geppert H, Vogt M, Bajorath J (2010). Current trends in ligand-based virtual screening: molecular representations, data mining methods, new application areas, and performance evaluation.. J Chem Inf Model.

[7] Jenkins JL, Kao RYT, Shapiro R (2003). Virtual screening to enrich hit lists from high-throughput screening: A case study on small-molecule inhibitors of angiogenin.. Protein Struct Funct Genet.

[8] Mestres J (2002). Virtual screening: a real screening complement to high-throughput screening.. Biochem Soc Trans.

[9] Shoichet BK (2004). Virtual screening of chemical libraries.. Nature.

[10] Wei L, Yu J (2008). Bioinformatics in China: A Personal Perspective.. PLoS Comput Biol.

[11] Kontijevskis A, Prusis P, Petrovska R, Yahorava S, Mutulis F (2007). A Look Inside HIV Resistance through Retroviral Protease Interaction Maps.. PLoS Comput Biol.

[12] Jenwitheesuk E, Horst JA, Rivas KL, Van Voorhis WC, Samudrala R (2008). Novel paradigms for drug discovery: computational multitarget screening.. Trends Pharmacol Sci.

[13] Yildirim MA, Goh KI, Cusick ME, Barabasi AL, Vidal M (2007). Drug-target network.. Nat Biotechnol.

[14] Paolini GV, Shapland RHB, van Hoorn WP, Mason JS, Hopkins AL (2006). Global mapping of pharmacological space.. Nat Biotechnol.

[15] Kitano H (2004). Biological robustness.. Nat Rev Genet.

[16] Keiser MJ, Roth BL, Armbruster BN, Ernsberger P, Irwin JJ (2007). Relating protein pharmacology by ligand chemistry.. Nat Biotechnol.

[17] Goh KI, Cusick ME, Valle D, Childs B, Vidal M (2007). The human disease network.. Proc Natl Acad Sci U S A.

[18] Wu CB, Ying H, Grinnell C, Bryant S, Miller R (2007). Simultaneous targeting of multiple disease mediators by a dual-variable-domain immunoglobulin.. Nat Biotechnol.

[19] Ricklin D, Lambris JD (2007). Complement-targeted therapeutics.. Nat Biotechnol.

[20] Schuster S, Fell DA, Dandekar T (2000). A general definition of metabolic pathways useful for systematic organization and analysis of complex metabolic networks.. Nat Biotechnol.

[21] Wishart DS (2008). DrugBank and its relevance to pharmacogenomics.. Pharmacogenomics.

[22] Bender A, Bojanic D, Davies JW, Crisman TJ, Mikhailov D (2008). Which aspects of HTS are empirically correlated with downstream success?. Curr Opin Drug Discov Dev.

[23] Sakharkar MK, Li P, Zhong ZW, Sakharkar KR (2008). Quantitative analysis on the characteristics of targets with FDA approved drugs.. Int J Biol Sci.

[24] Wist AD, Berger SI, Iyengar R (2009). Systems pharmacology and genome medicine: a future perspective.. Genome Med.

[25] Auffray C, Chen Z, Hood L (2009). Systems medicine: the future of medical genomics and healthcare.. Genome Med.

[26] Agoston V, Csermely P, Pongor S (2005). Multiple weak hits confuse complex systems: a transcriptional regulatory network as an example.. Phys Rev E Stat Nonlin Soft Matter Phys.

[27] Kitano H (2007). Innovation - A robustness-based approach to systems-oriented drug design.. Nat Rev Drug Discov.

[28] Stephen P, Vijayan R, Bhat A, Subbarao N, Bamezai RNK (2008). Molecular modeling on pyruvate phosphate dikinase of Entamoeba histolytica and in silico virtual screening for novel inhibitors.. J Comput-Aided Mol Des.

[29] Korcsmáros TS, Máté S, Böde C, Kovács IA, Csermely P (2007). How to design multi-target drugs.. Expert Opin Drug Discov.

[30] Stephanou A, McDougall SR, Anderson ARA, Chaplain MAJ (2005). Mathematical modelling of flow in 2D and 3D vascular networks: Applications to anti-angiogenic and chemotherapeutic drug strategies.. Math Comput Model.

[31] Spiro Z, Kovacs IA, Csermely P (2008). Drug-therapy networks and the prediction of novel drug targets.. J Biol.

[32] Qiao XB, Hou TJ, Zhang W, Guo SL, Xu XJ (2002). A 3D structure database of components from Chinese traditional medicinal herbs.. J Chem Inf Comp Sci.

[33] Tan F, Deng J (2002). Analysis of the constituents and antisenile function of Achyranthes bidentata polysaccharides.. Acta Bot Sin.

[34] Li Y, Xu C, Zhang QA, Liu JY, Tan RX (2005). In vitro anti-Helicobacter pylori action of 30 Chinese herbel medicines used to treat ulcer diseases.. J Ethnopharmacol.

[35] Satoh Y, Tashiro S, Satoh M, Fujimoto Y, Xu JY (1996). Studies on the bioactive constituents of Aurantii fructus immaturus.. Yakugaku Zasshi.

[36] Halgren TA (1996). Merck molecular force field. 1. Basis, form, scope, parameterization, and performance of MMFF94.. J Comput Chem.

[37] Adler M, Kochanny MJ, Ye B, Rumennik G, Light DR (2002). Crystal structures of two potent nonamidine inhibitors bound to factor Xa.. Biochemistry.

[38] Tucker TJ, Brady SF, Lumma WC, Lewis SD, Gardell SJ (1998). Design and synthesis of a series of potent and orally bioavailable noncovalent thrombin inhibitors that utilize nonbasic groups in the P1 position.. J Med Chem.

[39] Hopfner KP, Lang A, Karcher A, Sichler K, Kopetzki E (1999). Coagulation factor IXa: the relaxed conformation of Tyr99 blocks substrate binding.. Structure.

[40] Zbinden KG, Banner DW, Ackermann J, D'Arcy A, Kirchhofer D (2005). Design of selective phenylglycine amide tissue factor/factor VIIa inhibitors.. Bioorg Med Chem Lett.

[41] Olivero AG, Eigenbrot C, Goldsmith R, Robarge K, Artis DR (2005). A selective, slow binding inhibitor of factor VIIa binds to a nonstandard active site conformation and attenuates thrombus formation in vivo.. J Biol Chem.

[42] Tang J, Yu CL, Williams SR, Springman E, Jeffery D (2005). Expression, crystallization, and three-dimensional structure of the catalytic domain of human plasma kallikrein.. J Biol Chem.

[43] Huang MD, Syed R, Stura EA, Stone MJ, Stefanko RS (1998). The mechanism of an inhibitory antibody on TF-initiated blood coagulation revealed by the crystal structures of human tissue factor, Fab5G9 and TF center dot 5G9 complex.. J Mol Biol.

[44] Dullweber F, Stubbs MT, Musil D, Sturzebecher J, Klebe G (2001). Factorising ligand affinity: A combined thermodynamic and crystallographic study of trypsin and thrombin inhibition.. J Mol Biol.

[45] Emsley J, Cruz M, Handin R, Liddington R (1998). Crystal structure of the von Willebrand factor A1 domain and implications for the binding of platelet glycoprotein Ib.. J Mol Biol.

[46] Ngo JCK, Huang M, Roth DA, Furie BC, Furie B (2008). Crystal structure of human factor VIII: Implications for the formation of the factor IXa-factor VIIIa complex.. Structure.

[47] Papagrigoriou E, McEwan PA, Walsh PN, Emsley J (2006). Crystal structure of the factor XI zymogen reveals a pathway for transactivation.. Nat Struct Mol Biol.

[48] Everse SJ, Spraggon G, Veerapandian L, Doolittle RF (1999). Conformational changes in fragments D and double-D from human fibrin(ogen) upon binding the peptide ligand Gly-His-Arg-Pro-amide.. Biochemistry.

[49] Yee VC, Pedersen LC, Letrong I, Bishop PD, Stenkamp RE (1994). Three-Dimensional Structure of a Transglutaminase: Human Blood Coagulation Factor XIII.. Proc Natl Acad Sci U S A.

[50] Li XD, Hou TJ, Xu XJ (2005). Comparative studies of 14 binding free energies scoring functions.. Acta Phys-Chim Sin.

[51] Park H, Lee J, Lee S (2006). Critical assessment of the automated AutoDock as a new docking tool for virtual screening.. Proteins.

[52] Joshi-Tope G, Gillespie M, Vastrik I, D'Eustachio P, Schmidt E (2005). Reactome: a knowledgebase of biological pathways.. Nucleic Acids Res.

[53] Latora V, Marchiori M (2001). Efficient behaviour of small-world networks.. Phys Rev Lett.

[54] Csermely P, Agoston V, Pongor S (2005). The efficiency of multi-target drugs: the network approach might help drug design.. Trends Pharmacol Sci.

[55] Davidson BL (2003). Preparing for the new anticoagulants.. J Thromb Thrombolysis.

[56] Rai R, Sprengeler PA, Elrod KC, Young WB (2001). Perspectives on factor Xa inhibition.. Curr Med Chem.

[57] Di Nisio M, Middeldorp S, Buller HR (2005). Direct thrombin inhibitors.. N Engl J Med.

[58] Kranjc A, Kikelj D (2004). Dual inhibitors of the blood coagulation enzymes.. Curr Med Chem.

[59] Nishio H, Ieko M, Nakabayashi T (2008). New therapeutic option for thromboembolism—dabigatran etexilate.. Expert Opin Pharmacother.

[60] Guertin KR, Choi YM (2007). The discovery of the Factor Xa inhibitor otamixaban: from lead identification to clinical development.. Curr Med Chem.

[61] Ortolani O, Caggiano M, Mannelli R, Gogliettino A, Tufano R (1995). Protection from ischemia-reperfusion damage in patients with stroke: the role of rutin and GSH.. Transplant Proc.

[62] Krupinski K, Breddin HK, Giedrojc J, Bodzenta-Lukaszyk A, Bielawiec M (1995). Effects of 0-/-B-hydroxyethyl/rutoside on platelet function and thrombus formation in rat mesenteric vessels.. Mater Med Pol.

[63] Shi CS, Huang HC, Wu HL, Kuo CH, Chang BI (2007). Salvianolic acid B modulates hemostasis properties of human umbilical vein endothelial cells.. Thromb Res.

[64] Jiang R, Lau K, Hon P, Mak T, Woo K (2005). Chemistry and biological activities of caffeic acid derivatives from Salvia miltiorrhiza.. Curr Med Chem.

[65] Mitsos A, Melas IN, Siminelakis P, Chairakaki AD, Saez-Rodriguez J (2009). Identifying drug effects via pathway alterations using an integer linear programming optimization formulation on phosphoproteomic data.. PLoS Comput Biol.

