# Supplementary material for: A Network-Based Multi-Target Computational Estimation Scheme for Anticoagulant Activities of Compounds
Source: PLoS One. 2011 Mar 22;6(3):e14774. doi: 10.1371/journal.pone.0014774 (PMC3062543; doi:10.1371/journal.pone.0014774)
Supplement: Supporting Information S1 — (0.23 MB DOC) [file pone.0014774.s001.doc]

**Supporting Information S1**

**A Network-based Multi-Target Computational Estimation Scheme for Anticoagulant Activities of Compounds**

Qian Li^1,2,4,§^, Xudong Li^1,§^, Canghai Li^3^, Lirong Chen^1,*^, Jun Song^3^, Yalin Tang^2,*^, Xiaojie Xu^1,*^

^1^Beijing National Laboratory for Molecular Sciences, State Key Lab of Rare Earth Material Chemistry and Applications, College of Chemistry and Molecular Engineering, Peking University, Beijing, P.R. China.

^2^Beijing National Laboratory for Molecular Sciences, Center for Molecular Sciences, State Key Laboratory for Structural Chemistry of Unstable and Stable Species, Institute of Chemistry Chinese Academy of Sciences, Beijing, P.R. China.

^3^Experimental Research Center, China Academy of Chinese Medical Sciences, Beijing, P.R. China.

^4^Graduate University of Chinese Academy of Sciences, Beijing, P.R. China.

* E-mail: lirongchen@pku.edu.cn, tangyl@iccas.ac.cn, xiaojxu@pku.edu.cn

§ These authors contributed equally to this work.

**Part S1. 23 original plants and 1 original animal used in analysis**

Achyranthes bidentata, Angelica sinensis, Bupleurum chinensis, Carthamus tinctoria L., Citrus aurantii, Glycyrrhiza uralensis, Ligusticum wallichii, Paeonia rubra, Platycodon grandiflorum, Prunus persica, Rehmannia glutinosa, Salvia miltiorrhiza bge., Aromatica gaertner. f, Radix et rhizoma rhei, Radix paeoniae alba, Astragalus membranaeus (Fisch.)Bge., Polygonum cuspidatum, Lignum dalbergiae odoriferae, Campsis grandiflora, Hirudo, Panax notoginseng, Lignm sappan,

Rhizoma corydalis and Herba Leonuri.

The molecules used in the clotting assays were shown in Figure S1


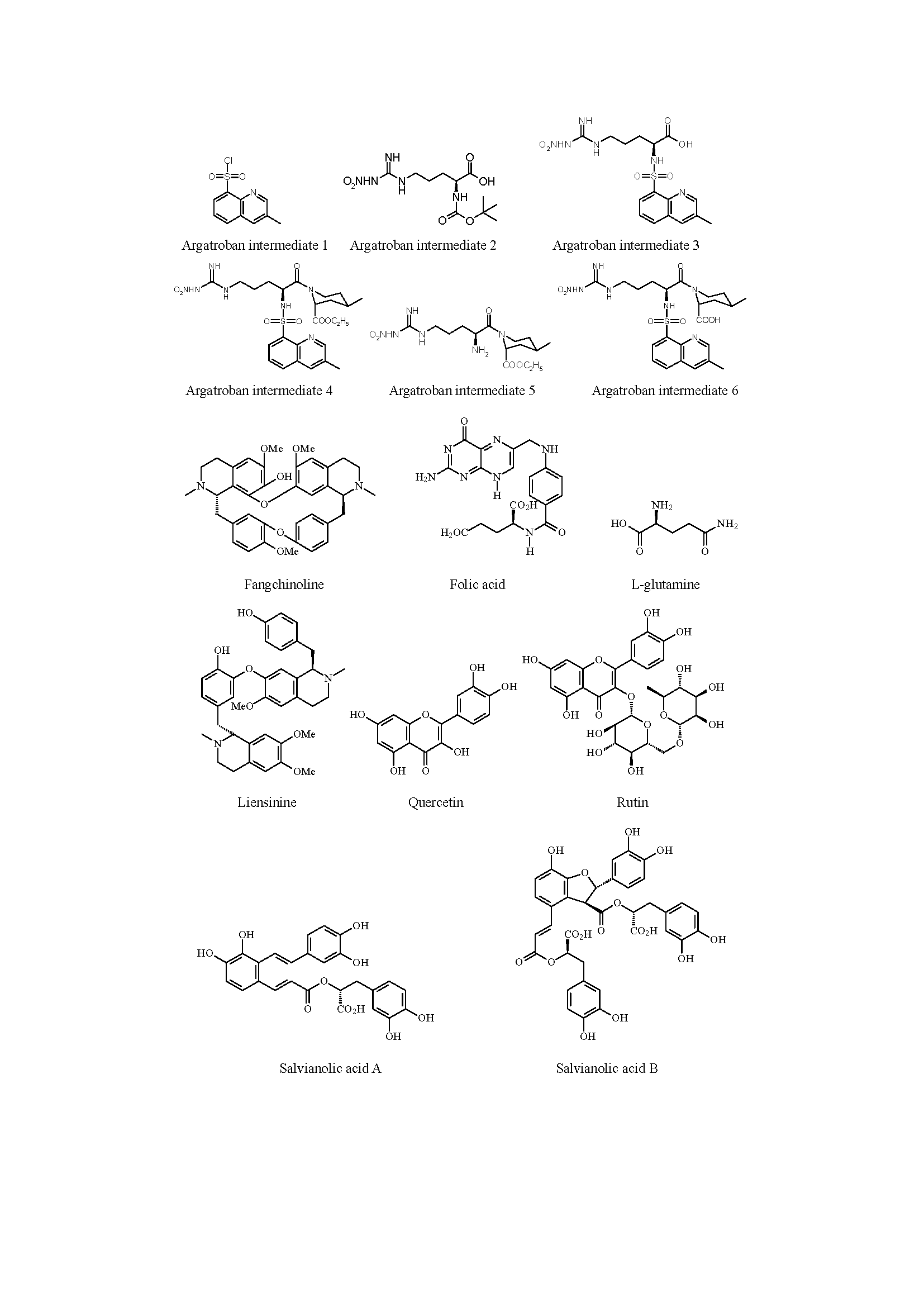


**Figure S1 The molecules used in the clotting assays.**

**Part S2. Verification of Predicting Reliability**

All overlays between experimental and computational conformations of five ligands are shown in the following figures. The docking results for coagulation factor Xa, thrombin, prothrombin and tissue factor/factor VIIa (Figure S2 (a-d)) are so satisfactory that all important chemical groups in predicted structures [coincide](http://dj.iciba.com/coincide/) with crystal ones, which can explain the reason of their smaller RMSDs well. For the ligand docked to factor VIIa, the chloride group orientes the different direction in the computational structure. The other reason that causes the bigger RMSD in factor VIIa docking is the reversals of a benzene ring and the amidino group. Other groups, however, superposes with experiment well, so the docking result is acceptable to our study. (Figure S2 (e)). The experimental conformations are showed in cyan and the docking ones are colored by atom type.


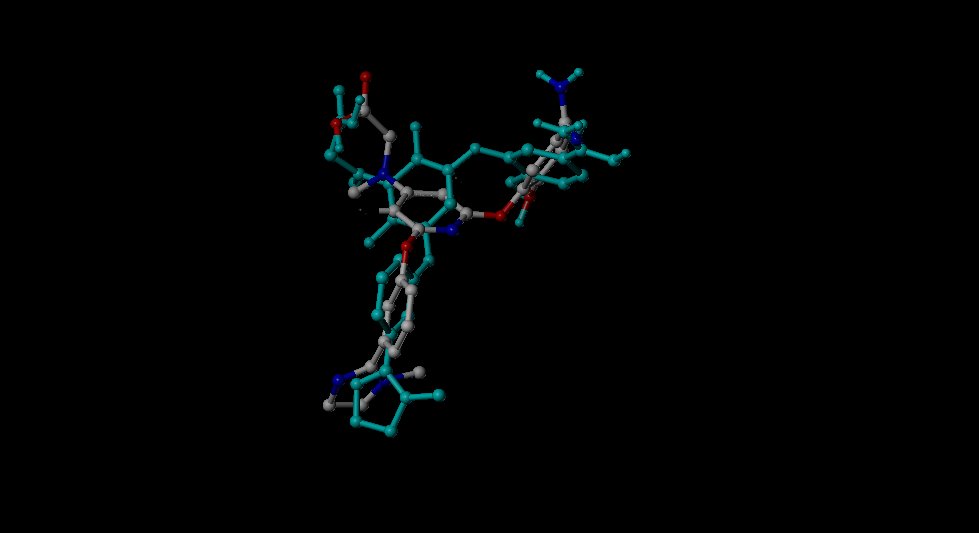


(a)


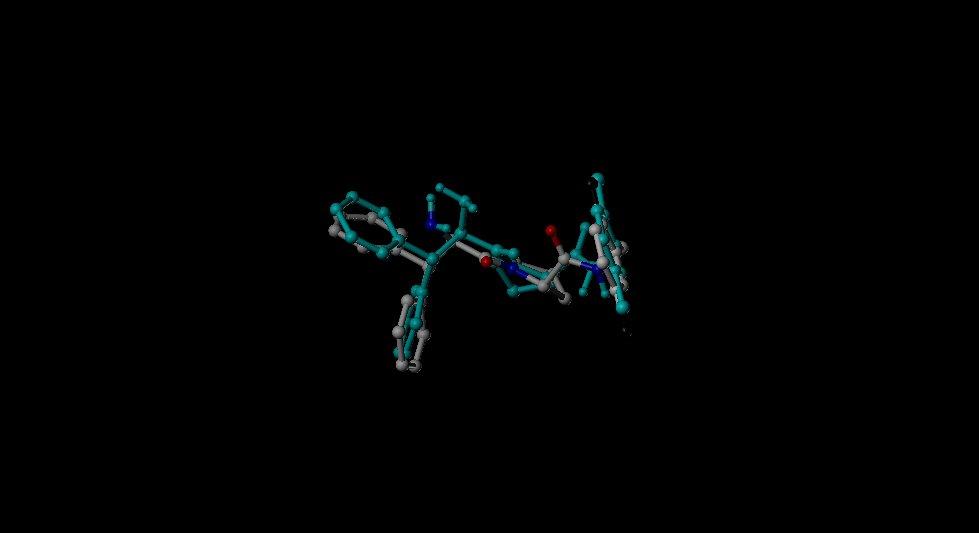


(b)


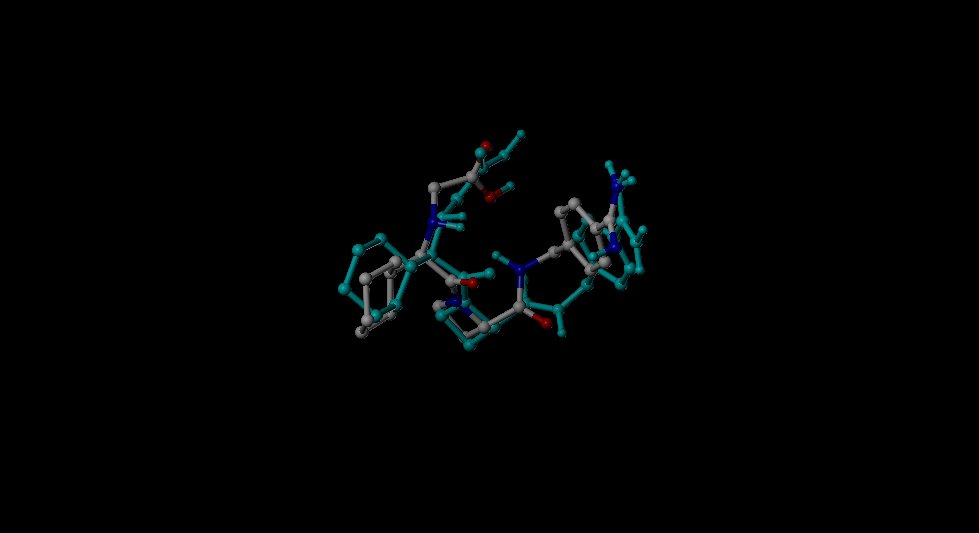


(c)


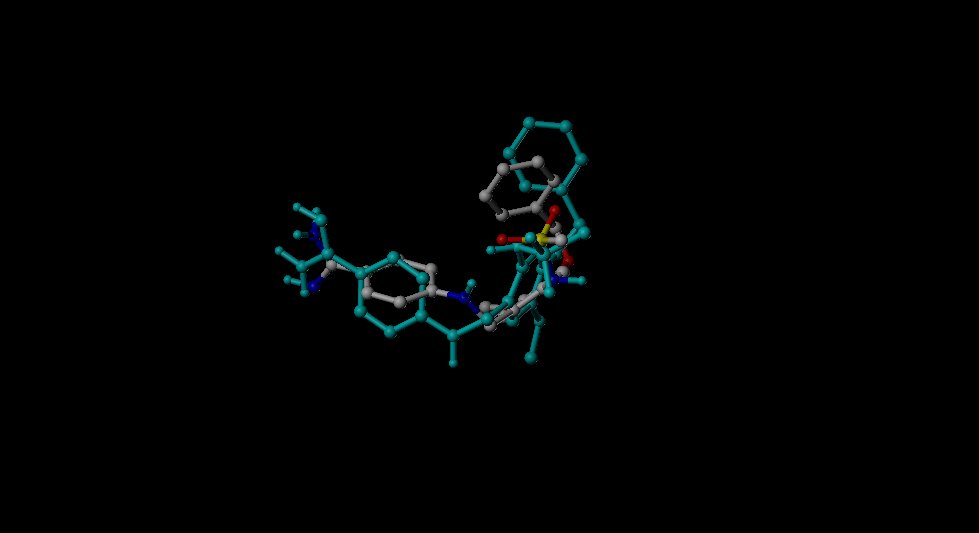


(d)


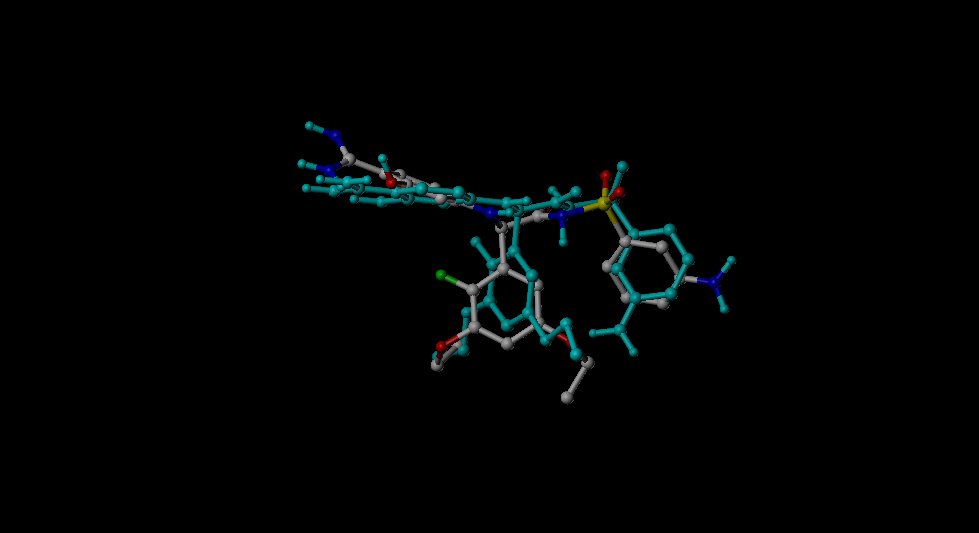


(e)

**Figure S2 Overlays between experimental and computational conformations of five ligands**

**Part S3. Experimental preparing and protocols**

**Chemicals**

Quercetin was purchased Sinopharm Chemical Reagent Co., Ltd. Rutin was ordered form Acros Organics. Salvianolic acid A, Salvianolic acid B, fangchinoline and liensinine were ordered from National Institute for the control of pharmaceutical and biological products. Six argatroban intermediates was purchased from Chemvon Chemical Reagent Co., Ltd. Unless noted otherwise, serum and regents were obtained from Steellex Scientific Instrument Company. Unless noted otherwise, serum and regents were obtained from Steellex Scientific Instrument Company. Coagulation assays were measured using [the Semi-Coagulation Analyzer](http://www.steellex.com/english/product_show.asp?column_cat_father_id=208&column_cat_id=214&top_id=208&strF=1) LG-PABER-I (4 channels). The photoelectrical electromagnetism measurement has been taken out in the coagulation testing.

**Pooled normal human plasma** (normal Hemostasis Reference Plasma): 10 x 1mL, freeze-dried. This product is prepared from a pool of Normal Human Plasma, collected in sodium citrate, and is rapidly processed, buffered and freeze-dried in a manner to assure the stability of hemostasis proteins. All analytes are in the NORMAL RANGE

**NORMAL (Abnormal) COAGULATION CONTROL PLASMA, (R2 Diagnostics, Inc.)**

Control should be run at the initiation of testing each day and at least once each shift, or with each group of assays. Controls should also be tested with each reagent change or major instrument adjustment. In laboratories where there is a heavy workload of PTs, and/or APTTs, test a normal and an abnormal control at a minimum of every 40 samples following protocols established for patient samples. Follow instructions provided by instrument and reagent manufacturers.

**One-Stage Prothrombin Time (PT) assay kit:**

The PT provides a functional determination of the integrity of the extrinsic (tissue factor) pathway of coagulation and is sensitive to the vitamin-K dependent clotting factors (factors II, VII, IX, and X) as well as to factors of the common pathway (fibrinogen, prothrombin, factor V, factor X). The PT is a widely used laboratory assay for the detection of inherited or acquired coagulation defects related to the extrinsic pathway of coagulation, and is the standard test for monitoring oral anticoagulation therapy.

**Activated Partial Thromboplastin Time (APTT) assay kit:**

The partial thromboplastin time (PTT) is the clotting time obtained when “partial thromboplastin” is added to plasma. Partial thromboplastin is the phospholipid fraction of a tissue extract, and differs from a complete tissue extract (i.e., “thromboplastin”) by the lack of tissue factor. The PTT is sensitive to the intrinsic pathway of coagulation, but is most sensitive to the contact factors (i.e., factor XII, prekallikrein, high molecular weight kininogen) when a particulate “activating agent” (i.e., silica, celite, kaolin, micronized silica, ellagic acid) is added to the reaction (activated PTT, aPTT). Many different phosophlipid reagents animal and plant origin, such as cephalin, have been used as partial thromboplastins, and a variety of activating substances are in use.

**Thrombin time(TT) assay kit:**

The thrombin time measures the thrombin-induced conversion of fibrinogen to fibrin directly in patient plasma, bypassing all other clotting factors. The thrombin time is performed by the addition of a low concentration of thrombin (usually bovine thrombin) directly to the citrated plasma and measuring the time required for the formation of fibrin monomers by visual, mechanical, or opto-electronic techniques. The thrombin time is prolonged by thrombin inhibitors and inhibitors of fibrin formation and polymerization, but it is not affected by problems with thrombin generation. Clinically, the thrombin time is often used to monitor heparin therapy, and to differentiate heparin effect, hypofibrinogenemia, dysfibrinogenemia, elevated levels of fibrin degradation products, and some paraproteins from other coagulopathies as the cause of a prolonged PT or aPTT. It is also used to monitor heparin reversal in following cardiothoracic surgery, and to monitor thrombolytic therapy. Increased plasma fibrinogen may also prolong the thrombin time, possibly by interfering with fibrin assembly. The reference range for the thrombin time is affected by the source and concentration of thrombin and other factors.

**Fibrinogen(Fg) assay kit:**

Fibrinogen is the most abundant clotting protein in the plasma, with a normal plasma level ranging rom 200-400 mg/dL. The quantitative determination of plasma fibrinogen is essential in the diagnosis and management of many coagulopathies. In addition, since plasma fibrinogen levels are increased in some patients who develop myocardial infarction and stroke, there is interest in the measurement of fibrinogen for thrombotic risk assessment. The washed clot method (total clottable fibrinogen assay, World Health Organization method) is the reference technique for fibrinogen determination. In this technique, citrated plasma is incubated for an extended.

**Clotting assays**

Activated partial thromboplastin time (aPTT)，prothrombin time(PT) and thrombin time(TT) assays were performed using a model LG-PABER-I coagulometer (Steellex Scientific Instrument Company, which also provided the used plasma and clotting reagents ) in compliance with manufacturers’ recommendations. All test chemicals with the exception of heparin sodium were solutized and subsequently serial ten-fold dilutions were prepared with dimethyl sulfoxide (DMSO) to yield a range of concentrations(12,1.2,0.12,and 0.012 ×10^-3^moles·L-1). Heparin sodium solutions (9.0×10^4^ I.U.·L-1) were prepared by dissolving in normal saline(NS) was used. The above series of solutions (DMSO and NS as references) were diluted with pooled normal human plasma (1:60 vol:vol), respectively , and the mixed plasma were evaluated with aPTT,PT,TT and Fg values, which associate with anticoagulant potential of test chemicals. All reactions were performed in duplicate and data is expressed in clot time (second).

**Experiment results**

Experiment results of APTT (Activated Partial Thromboplastin Time), PT (Prothrombin Time) and TT (Thrombin Time)

|  | APTT/s | PT/s | TT/s |
| --- | --- | --- | --- |
| **Physiological Saline** | **34.7** | **11.4** | **13.2** |
| salvianolic acid a | 38.9 | 13.4 | 17.1 |
| salvianolic acid b | 47.7 | 12.9 | 16.7 |
| **DMSO** | **45.6** | **12.3** | **14.3** |
| rutin | 48.2 | 11.3 | 25.5 |
| quercetin | 43.3 | 13.8 | 20.8 |
| liensinine | 55 | 13.8 | 20.7 |
| fangchinoline | 55.4 | 14.1 | 21.6 |
| folic acid | 57.8 | 13.9 | 21.7 |
| L-glutamine | 48.7 | 13.5 | 14.8 |
| Argatroban intermediate 1 | 63.1 | 13.1 | 15.9 |
| Argatroban intermediate 2 | 62.4 | 12.9 | 16.9 |
| Argatroban intermediate 3 | 56.7 | 13.3 | 16.1 |
| Argatroban intermediate 4 | 59.5 | 14.0 | 16.6 |
| Argatroban intermediate 5 | 53.2 | 12.7 | 17.1 |
| Argatroban intermediate 6 | 59.0 | 13.8 | 17.6 |

According to the solubility, salvianolic acid a and salvianolic acid b were dissolved with Physiological Saline. Other compounds such as rutin, quercetin, liensinine, fangchinoline, folic acid were dissolved with DMSO. Physiological saline and DMSO solution were treated as control solution to the two compound groups respectively. In the APTT and PT experiments, the concentrations of the compounds were 66.7uM. In the TT experiments, the concentrations of the compounds were 50uM.

The time increased by ratio compared to the control solution. Salvianolic acid a and salvianolic acid b compared to physiological saline and rutin, quercetin, liensinine, fangchinoline and folic acid compared to the DMSO solution. In order to show the integrated effect, we combined the data by sum the increased ratio of APTT, PT and TT together (the column “sum”).

|  | APTT | PT | TT | sum |
| --- | --- | --- | --- | --- |
| salvianolic acid a | 0.121 | 0.175 | 0.296 | 0.592 |
| salvianolic acid b | 0.375 | 0.132 | 0.265 | 0.771 |
| rutin | 0.057 | 0 | 0.783 | 0.84 |
| quercetin | 0 | 0.122 | 0.455 | 0.577 |
| liensinine | 0.206 | 0 | 0.113 | 0.319 |
| fangchinoline | 0.215 | 0.022 | 0.161 | 0.398 |
| folic acid | 0.268 | 0.007 | 0.167 | 0.442 |
| L-glutamine | 0.068 | 0.098 | 0.035 | 0.2 |
| Argatroban intermediate 1 | 0.384 | 0.065 | 0.112 | 0.56 |
| Argatroban intermediate 2 | 0.368 | 0.049 | 0.182 | 0.599 |
| Argatroban intermediate 3 | 0.243 | 0.081 | 0.126 | 0.45 |
| Argatroban intermediate 4 | 0.305 | 0.138 | 0.161 | 0.604 |
| Argatroban intermediate 5 | 0.167 | 0.033 | 0.196 | 0.395 |
| Argatroban intermediate 6 | 0.294 | 0.122 | 0.231 | 0.648 |

APTT (Activated Partial Thromboplastin Time), PT (Prothrombin Time), TT (Thrombin Time)

**Part S4. The network knock-out predictions**

Table S1

| The nodes which is knocked | The network efficiency |  | The nodes which is knocked | The network efficiency |
| --- | --- | --- | --- | --- |
| "factor Xa" | 8.894 |  | "factor XIIIa" | 16.867 |
| "thrombin" | 10.542 |  | "TF:VII" | 16.885 |
| "factor VIIIa:factor IXa" | 11.576 |  | "crosslinked fibrin multimer" | 16.948 |
| "factor IXa" | 13.146 |  | "prothrombin" | 17.099 |
| "factor XIa:GPIb:IX:V" | 13.448 |  | "factor VII" | 17.182 |
| "factor VIIIa" | 15.357 |  | "factor X" | 17.202 |
| "prothrombinase" | 15.560 |  | "kininogen" | 17.211 |
| "factor XIIa" | 15.585 |  | "vWF" | 17.245 |
| "TF:VIIa" | 15.719 |  | "sequestered TF" | 17.275 |
| "factor VIIa" | 15.790 |  | "factor VIII" | 17.345 |
| "fibrin" | 16.038 |  | "factor IX" | 17.371 |
| "cleaved factor XIII" | 16.038 |  | "factor V" | 17.384 |
| "factor Va" | 16.104 |  | "factor XII" | 17.420 |
| "kallikrein" | 16.134 |  | "factor XI" | 17.420 |
| "prekallikrein:kininogen:C1q bp" | 16.236 |  | "GPIb:IX:V" | 17.420 |
| "factor XI:GPIb:IX:V" | 16.286 |  | "prekallikrein" | 17.428 |
| "factor VIII:vWF" | 16.371 |  | "C1q bp" | 17.429 |
| "TF" | 16.610 |  | "fibrinogen" | 17.639 |
| "TFPI:Xa:TF:VIIa" | 16.751 |  | "factor XIII" | 17.639 |
| "kininogen:C1q bp" | 16.827 |  | "TFPI" | 17.722 |
| "fibrin multimer" | 16.867 |  | not any knock out | 17.822 |

Knock out of a single node and the effect to the network efficiency. We first calculated network efficiency for the whole network. Then knockout each target and calculated the relevant network efficiency. Knocking-out target make decrease of network efficiency and more decrease means more important of the relevant target.
